# Skilled Home Health Care Agency Perspectives on Communication With Physicians: A National Survey

**DOI:** 10.1093/geroni/igaa057.801

**Published:** 2020-12-16

**Authors:** Jonathan Norton, Amelie Nkodo, Bhavana Nangunuri, Danielle Pierotti, Kimberly Carl, Orla Sheehan, Cynthia Boyd, Bruce Leff

**Affiliations:** 1 Johns Hopkins University School of Medicine, Baltimore, Maryland, United States; 2 Eastern Virginia Medical School, Norfolk, Virginia, United States; 3 Visiting Nurse and Hospice for Vermont and New Hampshire, White River Junction, Vermont, United States; 4 Johns Hopkins Medicine, Baltimore, Maryland, United States

## Abstract

BACKGROUND: Communication is important in the care of older adults receiving skilled home health care (SHHC). In a prior national survey, physicians viewed communication and care coordination with SHHC agencies as dismal. The views of SHHC personnel (Registered Nurses, Licensed Practical Nurses, Physical Therapists, Occupational Therapists, and Speech-Language Pathologists) on this issue have not been well studied. OBJECTIVE: To determine the effectiveness of communication between SHHC personnel and physicians who order SHHC services. METHODS: A nationally representative mailed survey of personnel from SHHC agencies identified through the 2016 Home Health Compare data set from the Centers of Medicare and Medicaid Services. RESULTS: 263 of 2000 surveys returned (13.2% response rate). Responding agencies were mainly proprietary (75.3%) and urban-based (83.7%). Most agencies were in the South (38.8%); 28.3% Midwest, 22.9% West, 12.1% Northeast. Only 62.2% of SHHC personnel completing start of care visits (n=202) reported being able to contact a physician when needed. The most common strategies used to contact physicians are phone (76.0%) and fax (11.2%). The greatest barriers to communication are having to communicate through a third party (64.9%) and a perception by SHHC personnel that “Physicians [are] not interested in communicating with SHHC Personnel” (45.1%). Failed communication resulted in delayed orders (70.8%) and sending a patient to the emergency room (37.1%). IMPLICATIONS: SHHC agency personnel experience significant barriers in communicating with physicians. Modes of communication remain rudimentary, and there are serious consequences of failed communication.

